# In Vitro Comparison Between Different Types of Fissure Sealants in Permanent Teeth—Micro-CT Study

**DOI:** 10.3390/polym18141790

**Published:** 2026-07-22

**Authors:** Shara I. Sajini, Jehad Al-Mutiri, Fares Al-Harbi, Mai Almarzouki

**Affiliations:** 1 Department of Restorative Dentistry, Faculty of Dentistry, King Abdulaziz University, Jeddah 21589, Saudi Arabia; mzalmarzouki@kau.edu.sa; 2 Faculty of Dentistry, King Abdulaziz University, Jeddah 21589, Saudi Arabia; jehad.almutiri@gmail.com (J.A.-M.); fares.alharbi9966@gmail.com (F.A.-H.)

**Keywords:** fissure sealant, adaptation, microleakage, bioactive, micro-CT

## Abstract

Occlusal pits and fissures remain the most vulnerable tooth surfaces for caries initiation, and selecting a sealant that seals these areas reliably is a clinically meaningful question. We investigated the internal adaptation of four pit and fissure sealants—two resin-based and two glass ionomer-based—applied to permanent premolar teeth. Forty extracted premolars were randomly assigned to four groups (*n* = 10): Group 1, BeautiSealant (BS); Group 2, 3M Clinpro Sealant; Group 3, GC Fuji IX without surface coating; and Group 4, GC Fuji IX with G-Coat Plus. Following 15,000 thermal cycles, internal adaptation was assessed by X-ray microtomography (micro-CT) using silver nitrate as a tracer. No statistically significant difference was found in the overall mean gap volumes between groups (*p* = 0.774). The 3M™ Clinpro™ Sealant group exhibited the lowest mean gap value (3.45 ± 1.71), followed by BeautiSealant (3.89 ± 1.34) and GC Fuji IX GP^®^ with G-Coat Plus™ (4.93 ± 4.09), whereas the GC Fuji IX GP^®^ without coating group showed the highest mean value (5.56 ± 5.35). Resin-based sealants achieved better fissure penetration and marginal adaptation than the glass ionomer formulations tested, though the differences did not reach statistical significance under the conditions of this study.

## 1. Introduction

Dental caries ranks among the most prevalent chronic diseases worldwide, arising from the interplay between cariogenic biofilm, host factors, and dietary sugars, all of which act on a tooth surface whose natural anatomy can either facilitate or resist plaque accumulation [1]. From a minimally invasive standpoint, sealing pits and fissures before caries establishes itself is arguably the most straightforward preventive strategy available, as it physically excludes the substrate–microorganism interaction that drives early lesion formation [2]. Mechanically, sealants achieve retention through microtags formed within acid-etched enamel, creating a barrier against food debris and bacteria on the occlusal surface and thereby halting both initiation and progression of caries [3]. The uptake of this approach has grown considerably: comparing (NHANES) data from 2011 to 2012 with earlier figures from 1999 to 2004 revealed sealed permanent teeth in roughly 31% of 6–8-year-olds, 49% of 9–11-year-olds, and 43% of adults [4,5].

Sealant materials fall broadly into three compositional categories: resin-based fissure sealants (RBS), glass ionomer (GI) sealants, and polyacid-modified resin sealants, each further subdivided by opacity or, in the case of glass ionomers, by whether they incorporate a resin phase [6,7,8]. Neither family is without shortcomings. Resin-based formulations are sensitive to moisture contamination during placement and undergo polymerization shrinkage, both of which can open marginal gaps and limit use in partially erupted teeth [9]. A tendency toward a more resistant biofilm on resin surfaces has also been reported. Conventional glass ionomer, meanwhile, bonds chemically to enamel and releases fluoride, but its relatively poor mechanical properties make it prone to fracture under occlusal loading when used in load-bearing fissures [10]. Distinct clinical advantages and limitations are provided by various classes of fissure sealants. Resin-based sealants typically exhibit superior retention and deeper fissure penetration due to their lower viscosity and micromechanical bonding to etched enamel. In contrast, glass ionomer sealants provide fluoride release, chemical adhesion, and a greater tolerance to moisture contamination, which may be advantageous in partially erupted teeth or when ideal isolation is challenging. However, glass ionomer materials tend to have lower retention and may be more technique- and material-dependent in long-term performance. Consequently, the clinical relevance of comparing these materials is preserved, particularly when evaluating their sealing ability, retention, fluoride release, and handling characteristics across various clinical settings [11].

Previous comparative studies have shown that the key differences between fissure sealants may be found in their clinical performance profiles, rather than in the prevention of overall caries. The caries preventive effect of glass ionomer and resin-based fissure sealants was not statistically significant in a meta-analysis by Yengopal et al., indicating that both materials can be used clinically for occlusal caries prevention [11]. Additionally, Mickenautsch and Yengopal’s revised systematic review failed to identify any definitive evidence favouring one material over another for caries prevention [12]. However, the authors acknowledged that the selection of materials may still be influenced by practical clinical considerations, including moisture control, handling characteristics, and the clinical scenario. Taken together, these findings indicate that resin-based and glass ionomer sealants may have equivalent preventative outcomes, despite variations in retention, tolerance to moisture, and material behaviour.

The concept of bioactivity in dental materials refers to the capacity of a material to interact biologically with host tissues, typically by releasing ions that modulate cellular activity or promote hydroxyapatite precipitation on dental hard tissue surfaces [13]. A small number of bioactive sealants have reached the market; these materials are designed to respond dynamically to pH fluctuations by releasing and subsequently recharging fluoride and other ionic components, theoretically addressing the clinical weaknesses of earlier generations. Among the practical demands placed on any sealant is adequate flow viscosity: the material must reach the depths of narrow, irregular fissures before setting [14]. Once placed, long-term success depends heavily on marginal integrity, since even minor gaps along the sealant periphery allow ingress of bacteria and substrate, ultimately leading to material dissolution and secondary caries [14,15].

Micro-computed tomography has emerged as a valuable non-destructive tool for quantifying internal and marginal adaptation, replacing or complementing traditional serial sectioning. Unlike dye penetration methods, micro-CT captures three-dimensional gap volumes across the entire specimen without altering its structure, and advances in reconstruction software now allow fine-grained analysis of polymerization dynamics in dental resins [16,17]. The growing variety of bioactive sealant formulations, combined with the limitations of earlier evaluation methods, makes a systematic comparison of their adaptation behavior both timely and clinically relevant [18]. Compared with conventional leakage and sectioning techniques, micro-CT offers a more comprehensive view of gap distribution and is especially useful when comparing materials with different viscosities and setting mechanisms. Although previous studies have investigated fissure sealants using micro-CT and related methods, direct comparisons among contemporary resin-based, giomer-based, and coated versus uncoated glass ionomer protocols under standardized conditions remain limited. Therefore, the present in vitro study compared the internal adaptation of four commercially available fissure sealant protocols in permanent premolars using micro-CT. The null hypothesis was that sealant composition would not influence gap formation at the sealant–tooth interface.

## 2. Materials and Methods

### 2.1. Ethical Approval and Specimen Selection

All procedures were carried out in accordance with the Declaration of Helsinki and received prior approval from the King Abdulaziz University Ethical Committee (Approval No. 153-12-20). Forty intact human permanent premolars were collected following routine orthodontic extractions. Only teeth free from visible caries, cracks, or restorations were included. Written informed consent was obtained from adult donors; for patients under 18, consent was provided by a parent or legal guardian. For this in vitro comparison study, a balanced sample of 40 premolars was selected to allow equal allocation across the four study groups (*n* = 10 per group), in line with the scale of comparable laboratory micro-CT investigations.

### 2.2. Specimen Preparation

Prior to sealant application, the extracted premolars were debrided of residual soft-tissue deposits using a rotating prophylaxis brush and pumice slurry to remove surface biofilm, and then stored in normal saline until the experimental procedures were initiated. All specimen-preparation procedures were carried out by two calibrated operators simultaneously, using a predefined standardized protocol to reduce technique-related variability. The root apices were sealed with composite resin and covered with wax. The crowns of the teeth were coated with nail varnish, leaving a uniform 1 mm margin around the sealed fissure that was exposed. The exposed window was standardized using a periodontal probe under direct visual inspection. The occlusal surfaces were then cleaned with a prophylaxis brush and pumice slurry, rinsed thoroughly, and air-dried to standardize the enamel surface condition prior to treatment. During these procedures, the teeth were secured in a specimen holder to maintain consistent orientation and handling.

Specimens were allocated to four groups (*n* = 10) by simple random assignment according to the sealant protocol. Group 1 (BeautiSealant, Shofu)*:* a self-etch primer was applied to the enamel for 5 s and gently air-dried. BeautiSealant was dispensed through a syringe needle, spread with a microbrush, and light-cured for 20 s. Group 2 (Clinpro, 3M ESPE)—enamel was conditioned with Scotchbond Universal Etchant (37% phosphoric acid; 3M, St. Paul, MN, USA) for 15 s, rinsed for 10 s, and blotted dry with a cotton pellet. Clinpro Sealant was applied and light-cured for 20 s. Group 3 (GC Fuji IX GP^®^ without coating)—GC Cavity Conditioner was applied with a microbrush for 15 s, then rinsed and air-dried. GC Fuji IX GP^®^ was packed into the fissure with a microbrush and allowed to self-cure for approximately 2.5 min. Group 4 (GC Fuji IX GP^®^ + G-Coat Plus): the same placement protocol as in Group 3 was followed, and G-Coat Plus was then immediately applied and light-cured for 20 s. Full material compositions are listed in Table 1.

### 2.3. Thermal Cycling and Silver Nitrate Staining

Specimens were thermocycled between 5 °C and 55 °C for 15,000 cycles (30 s dwell per bath; 15 s transfer time; Thermocycler THE-1100, SD Mechatronik, Feldkirchen-Westerham, Germany). This protocol is widely accepted as a reasonable simulation of intraoral thermal fatigue. Thermocycled specimens were immersed in ammoniacal silver nitrate solution in the dark for 8 h—prepared by dissolving silver nitrate in distilled water and titrating with ammonium hydroxide to produce the ammoniacal complex—then rinsed under running water and placed in a photo-developing solution under fluorescent light for a further 8 h. Reduced silver deposits mark the extent of tracer penetration.

### 2.4. Micro-CT Imaging and Analysis

**Figure 1:**
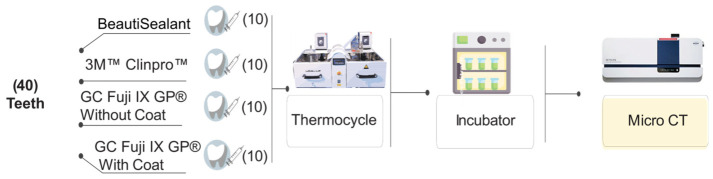
Schematic illustration of the study design and material allocation.

A desktop high-resolution micro-CT unit (Model 1172, Skyscan/Bruker, Kontich, Belgium) was used for all image acquisitions. Scanning parameters were: 100 kV, 98 μA, 1 mm aluminum filter, 180° rotation in 0.40° increments, gain 1.0, exposure 3.7 s. Cone-beam reconstruction was performed in NRecon (Skyscan), and volumetric analysis in CT-Analyser v1.15 (Skyscan), using a standardized workflow applied consistently to all specimens. The outcome variable was total gap volume, the void between the tooth surface and sealant quantified as the volume of AgNO3 penetration per specimen, expressed as a percentage. A schematic illustration of the study design and material allocation is presented in Figure 1.

### 2.5. Statistical Analysis

Group data are presented as mean ± standard deviation, with median values and 95% confidence intervals additionally reported to support interpretation. Normality was assessed using the Shapiro–Wilk test. Inter-group comparisons were performed using the Kruskal–Wallis test as a conservative non-parametric approach for this modest exploratory sample. The corresponding test statistic and an effect-size estimate were considered when interpreting the findings. All analyses were performed in SPSS v25.0 (IBM, Chicago, IL, USA), and *p* ≤ 0.05 was considered statistically significant.

## 3. Results

The microleakage values showed numerical differences among the four tested sealant protocols; however, these differences were not statistically significant. The 3M™ Clinpro™ Sealant group exhibited the lowest mean microleakage value (3.45 ± 1.71), followed by BeautiSealant (3.89 ± 1.34) and GC Fuji IX GP^®^ + GCoat Plus™ (4.93 ± 4.09), whereas the GC Fuji IX GP^®^ without coating group showed the highest mean value (5.56 ± 5.35). Descriptive statistics, including median values and 95% confidence intervals, are presented in Table 2. The overall comparison among groups was not significant (Kruskal–Wallis H = 1.11, *p* = 0.774), and the estimated effect size was negligible (ε^2^ = 0.00). Accordingly, although resin-based materials showed numerically lower gap values, the findings should be interpreted cautiously and do not support a statistically significant inter-material difference under the present experimental conditions.

**Figure 2:**
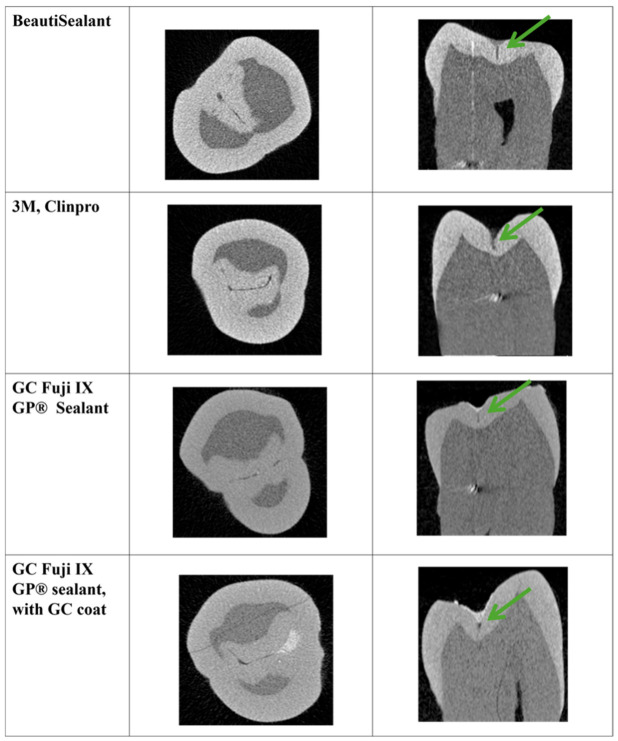
Representative micro-CT images of the tested sealant groups showing interfacial gap formation; arrows indicate representative regions of interest at the tooth–sealant interface.

Representative micro-CT images shown in Figure 2 demonstrated interfacial gap formation in all groups, although the extent of the gaps varied. The 3M™ Clinpro™ Sealant group showed comparatively smaller and less conspicuous gaps, while the GC Fuji IX GP^®^ without coating group exhibited more pronounced gap formation along the tooth–sealant interface. The BeautiSealant and G-Coat Plus™ groups presented intermediate findings, with visible interfacial discrepancies but without the same degree of gap extension observed in the uncoated glass ionomer group. Overall, the quantitative and imaging findings suggest a more favorable sealing pattern for the resin-based sealant, although this trend did not reach statistical significance.

## 4. Discussion

Occlusal surfaces account for only about 12% of the total tooth surface area, yet they harbour 67–90% of all carious lesions in children—a disparity that reflects the anatomical complexity of pits and fissures and the difficulty of removing plaque from them [19]. Sealants work by eliminating the ecological niche that supports cariogenic biofilm, and their effectiveness is well established when retention is maintained; the challenge lies in achieving and sustaining an adequate seal across materials with different chemical and mechanical properties [19,20,21]. Enamel adhesion is the foundation of sealant performance: without a reliable bond, marginal gaps develop, bacteria infiltrate, and secondary caries follows—outcomes that ultimately compromise both the sealant and the tooth [22,23,24]. The present study examined this question directly by quantifying gap formation in four contemporary sealant systems using micro-CT, which we chose specifically because it allows non-destructive, three-dimensional assessment of the entire sealant–enamel interface rather than a limited number of cross-sections [25].

Premolars extracted for orthodontic reasons were selected because they are structurally uniform, relatively free from pre-existing structural variation, and available without imposing additional risk on patients [23,26]. Thermal cycling was used to simulate the cumulative fatigue of intraoral temperature fluctuations; repeated expansion and contraction cycles stress the sealant–enamel interface, and any weakness in adaptation tends to manifest as measurable gap formation [27,28]. One methodological consideration is that glass ionomer materials are intrinsically hydrophilic, which means conventional dye penetration studies may overestimate microleakage due to nonspecific dye absorption [29]. Using silver nitrate and micro-CT largely avoids this confound, and this choice likely explains why our glass ionomer results are more moderate than some dye-based reports in the literature.

Our primary finding—that no statistically significant difference existed among groups—leads us to accept the null hypothesis. In practical terms, this means that under the conditions tested, all four sealants performed comparably in terms of gap formation, despite their very different compositions. This is broadly consistent with Hatirli et al., who also found equivalent sealing performance between resin-based and glass ionomer bioactive sealants following cyclic thermo-mechanical simulation [30]. The data from Demirel et al., using a similar micro-CT approach, reached the same conclusion [31]. Clinpro nonetheless showed the lowest numerical mean gap volume among the four groups, which aligns with the established understanding that resin-based sealants—when placed under optimal isolation conditions—form more intimate contact with acid-etched enamel [32,33].

BeautiSealant, a giomer incorporating S-PRG fillers, performed similarly to Clinpro. The S-PRG particle technology confers both fluoride cycling capability and moisture tolerance, which is particularly relevant in partially erupted teeth where perfect isolation is difficult to achieve [24,34]. These properties make giomers clinically attractive even if the present data do not show a measurable adaptation advantage over conventional resin sealants. The chemical adhesion of glass ionomers to enamel is frequently cited as a benefit, and the ion-releasing properties are genuinely useful cariologically [31]. However, glass ionomer formulations are generally more viscous and therefore less able to penetrate deep, narrow fissures compared with low-viscosity resins, and their comparatively weaker mechanical properties may explain the trend toward higher gap values seen in Group3 [35,36,37]. This pattern—higher mean gaps for uncoated GIC, lower for coated GIC—is consistent with reports showing that G-Coat Plus reduces early moisture sensitivity and surface dissolution of glass ionomer [38,39,40,41].

The recommendation to protect freshly placed glass ionomer with a hydrophobic coating is not new [39,42], but our findings reinforce it specifically in the context of fissure sealing. The mean gap volume in Group4 was notably lower than Group 3, suggesting that G-Coat Plus meaningfully reduces early water contamination during the initial setting phase—even if this advantage did not produce statistical significance at our sample size. Clinically, this implies that glass ionomer sealants should not be used without an immediate protective coating, particularly in high-caries-risk patients or where isolation is imperfect. Several limitations should be acknowledged when interpreting the results of the present investigation. The small sample size may have resulted in diminished statistical sensitivity to detect slight inter-material changes. In addition, the study design could not completely simulate the biological, chemical and mechanical intricacies of the oral environment, including the impact of saliva, occlusal loading, pH variations and biofilm activity, as it was an in vitro study. Variations in fissure morphology among various tooth types may potentially affect the generalizability of the results, as the analysis was restricted to extracted premolars. In addition, the micro-CT measurements were not formally assessed for repeatability and reproducibility. Although all specimens were analyzed with the same imaging technology and a consistent analytical procedure, the lack of a specific reliability study should be mentioned when addressing the precision and consistency of the quantitative values. Also, material retention was not assessed, and hence the current findings should be viewed as indicative of internal adaptability and microleakage, and not as a representation of long-term clinical efficacy or sealant longevity. Another crucial factor is the possible effect of the viscosity of the material on the fissure penetration and the interfacial adaptation. Viscosity differences may alter the capacity of a sealant to flow into narrow or anatomically complex fissures, which may affect gap creation and sealing behavior. Lower viscosity materials tend to have better penetration and better adaptation, while higher viscosity materials tend to be more sensitive to inadequate adaptation in limited fissure areas. Because viscosity was not measured directly in the present study, its contribution to the observed numerical differences remains inferential and should be interpreted with caution. Although thermocycling is the most frequently employed artificial aging technique, it is unable to replicate all aspects of long-term intraoral degradation. Finally, operator-dependent variability during specimen preparation was not analysed separately, although all procedures were performed according to a standardized laboratory workflow. Future research that includes experimental evaluation of viscosity-related behavior under clinically relevant aging conditions, formal measurement-reliability analysis, direct retention assessment, and larger samples would further strengthen the evidence base in this field.

## 5. Conclusions

Under the conditions of this in vitro study, all four sealants produced comparable levels of microleakage, and no material demonstrated a statistically significant advantage. Numerically, Clinpro achieved the lowest gap volumes, and GC Fuji IX, without coating, the highest. Within the limitations of the present study, resin-based materials appeared to provide more favourable adaptation, while glass ionomer-based sealants may benefit from immediate hydrophobic surface protection to reduce early dissolution and marginal compromise.

## Figures and Tables

**Table 1:** Composition of the materials used in this study.

Material Class	Commercial Product (Manufacturer)	Composition
Resin-modified, fluoride-releasing fissure sealant	BeautiSealant (Shofu, Kyoto, Japan)	S-PRG fillers, TEGDMA, UDMA
Conventional resin-based fissure sealant	3M™ Clinpro™ Sealant (3M ESPE, St. Paul, MN, USA)	Bis-GMA, TEGDMA, EDMAB, BHT, TBATFB
Glass ionomer-based fissure sealant	GC Fuji IX GP^®^ (GC Corporation, Tokyo, Japan)	Powder: alumino-fluoro-silicate glass; Liquid: polyacrylic acid
Coating varnish	G-Coat Plus™ (GC Corporation, Tokyo, Japan)	HEMA, Bis-GMA, TEGDMA, CQ, fluoride, additives

**Table 2:** Descriptive statistics for microleakage (%) across the four sealant groups.

Material	Mean (%)	SD	Median (%)	95% CI of Mean	Overall *p*-Value
BeautiSealant (Group 1)	3.89	1.34	4.22	2.93–4.85	0.774
3M™ Clinpro™ (Group 2)	3.45	1.71	3.27	2.23–4.67	—
GC Fuji IX GP^®^ (Group 3)	5.56	5.35	3.52	1.73–9.39	—
GC Fuji IX + G-Coat Plus™ (Group 4)	4.93	4.09	3.12	2.00–7.86	—

## Data Availability

Data supporting the reported results are available upon reasonable request from the corresponding author.

## Abbreviations

The following abbreviations are used in this manuscript:

GIC	Glass ionomer cement
S-PRG	Surface-pre-reacted glass ionomer
Micro-CT	Micro-computed tomography
RBS	Resin-based sealant
